# Bayesian Learning of Shifted-Scaled Dirichlet Mixture Models and Its Application to Early COVID-19 Detection in Chest X-ray Images

**DOI:** 10.3390/jimaging7010007

**Published:** 2021-01-10

**Authors:** Sami Bourouis, Abdullah Alharbi, Nizar Bouguila

**Affiliations:** 1 Department of Information Technology, College of Computers and Information Technology, Taif University, Taif, P.O. Box 11099, Taif 21944, Saudi Arabia; amharbi@tu.edu.sa; 2 The Concordia Institute for Information Systems Engineering (CIISE), Concordia University, Montreal, QC H3G 1T7, Canada; nizar.bouguila@concordia.ca

**Keywords:** infection detection, COVID-19, X-ray images, image classification, bayesian inference, shifted-scaled dirichlet distribution, MCMC, gibbs sampling

## Abstract

Early diagnosis and assessment of fatal diseases and acute infections on chest X-ray (CXR) imaging may have important therapeutic implications and reduce mortality. In fact, many respiratory diseases have a serious impact on the health and lives of people. However, certain types of infections may include high variations in terms of contrast, size and shape which impose a real challenge on classification process. This paper introduces a new statistical framework to discriminate patients who are either negative or positive for certain kinds of virus and pneumonia. We tackle the current problem via a fully Bayesian approach based on a flexible statistical model named shifted-scaled Dirichlet mixture models (SSDMM). This mixture model is encouraged by its effectiveness and robustness recently obtained in various image processing applications. Unlike frequentist learning methods, our developed Bayesian framework has the advantage of taking into account the uncertainty to accurately estimate the model parameters as well as the ability to solve the problem of overfitting. We investigate here a Markov Chain Monte Carlo (MCMC) estimator, which is a computer–driven sampling method, for learning the developed model. The current work shows excellent results when dealing with the challenging problem of biomedical image classification. Indeed, extensive experiments have been carried out on real datasets and the results prove the merits of our Bayesian framework.

## 1. Introduction and Related Works

Pneumonia is a severe disease issue resulting in inflammation of the lungs where a large number of people lose their lives every day. The causes of this infectious disease could be attributed to viruses or bacteria. Today, the SARS-CoV-2 virus named COVID-19 pneumonia is causing a significant outbreak around the world, having a serious impact on the health and life of several people. In particular, it causes pneumonia in humans and carries severe infections between people. Patients with COVID-19 can have acute symptoms and some may die of major organ failure. One of the critical steps in the fight against this disease is the possibility to quickly detect and track contaminated persons and place them under particular care. Early inspection of confirmed cases is of great urgency because of its infectious nature. One of the many ways of detecting the disease is by a chest radiographs of the patient. Recently, some studies have shown that studying COVID-19 from Chest X-ray images may be considered as the quickest solution to diagnose patients [1]. It is noteworthy that chest X-ray radiography is one of the interesting imaging to diagnose several related chest diseases such as pneumonia, lung cancer, emphysema and pulmonary edema [2,3]. However, sometimes this medical imaging can be subject to error for inexperienced radiologists, while being tedious for experienced ones. Visual examination of these radiographs is generally restricted due to low infectious disease specificity. In addition, the presence of noise, the contrast which is often insufficient between the soft tissues and the overlap in appearance properties are often sources of error for an accurate diagnosis [1,4]. These inconsistencies can result in important biased decisions for clinicians.

To deal with these drawbacks and to detect infected patients, it is necessary to develop effective and automated computerized support tools able to offer radiologists desirable measures about the disease severity. These tools should also allow rapid detection and prediction of any possible infection, in particular COVID-19. Nevertheless, performing a precise analysis of big biomedical data is too difficult and time consuming because these images contain various patterns and symptoms at different stages (early, middle, advanced) [4,5]. For instance at the early stage, it is not easy at all to discover COVID-19 symptoms having acute respiratory distress syndrome in chest X-ray (CXR) scans because these symptoms can look similar to other viral infections like RSV pneumonia. Consequently, it is important to consider such assumption and to take into account robust features extraction techniques when implementing new systems.

Several promising algorithms have been implemented in the past decades to deal especially with infection detection. Some traditional machine learning-based methods are applied to support pneumonia diagnosis in children by classifying chest radiographs into normal or pneumonia cases [6]. Haar wavelet transform is also investigated as an effective feature extraction technique. Some classifiers such as FCM, DWT and WFT [2] and K-nearest neighbor (k-NN) [3] were exploited in this context to detect pneumonia infection. Nevertheless, these conventional methods fail to identify properly lung with lesions. It is true that traditional methods helped the specialists in their diagnosis, but the resulting accuracy was poor. Thus, other image processing-based systems have been proposed to address the problems of infection localization and detecting malicious lesions using, for example, SVM, Neural Networks (NN) and Deep NN (DNN) [5,7,8,9].

The Fully CN (FCN) method is also applied for segmenting lung in CXR [10]. Another work which is conducted using deep learning method is proposed in [11] to classify CT scan and chest X-ray into three classes: influenza-A viral pneumonia, COVID-19, and normal. The obtained accuracy is 89.3%. As a result, the accuracy is 89.3% and the training process takes a long time. After studing the related work, it is obvious that the success of supervised CNN and deep learning methods to classify CXR images and detect COVID-19 relies mainly on the size of training data. For smaller data set, these techniques are not suitable since this size is responsible for poor performances and in many cases, it becomes too difficult to generate more training data. Thus, it is important to look for other alternatives. Features extraction methods are also exploited in conjuction with some classifiers in order to extrcat ans select relevant visual features. For instance, the ResNet50 feature extractor is used with SVM and CNN for detecting and classifying lung nodule disease in chest CT- images [12]. Other approaches such as registration and active shape models [13,14] are exploited with pixel-based statistical classification methods in order find the boundary/region targets. For example, the lung region is determined through a non-rigid registration step between the chest radiograph of the image patient and a reference model [13].

The good results obtained from applying artificial intelligence and machine learning models to some previous epidemics are motivating researchers to provide new perspective for addressing this novel coronavirus outbreak. In particular, classifying non-Gaussian data in an unsupervised way can be of great interest for automated medical applications. Among the main existing methods to tackle this problem, statistical mixture models have recently gained considerable interest from both the theoretical and practical points of view [15,16,17,18,19,20]. This approach has led to the design of new more efficient tools. Our work is mainly based on recent research findings that have shown modeling visual data (such as images) effectively is very important for further applications such as image classification. In particular, the taking into account of the distribution of Dirichlet is very interesting to deal with non-Gaussian data modelling [21]. Other derived models such as the scaled Dirichlet mixture (a generalization of the Dirichlet) [16] have also been shown to be effective for data grouping and classification. Further works have show that it is possible to improve these last two models by introducing an additional parameter which leads to a more flexible model. The resulting statistical mixture is called shifted-scaled Dirichlet mixture (SSDMM) and is assumed to be a generalization of the scaled model (here the Shifted term mean a perturbation in the simplex). This new model has been applied successfully for a variety of applications [22].

## 2. Motivations

The work developed in [22] is based on a shifted-scaled Dirichlet mixture model (SSDMM) and evaluated for data clustering and writer identification. Two important issues arise when deploying mixture models which are calculating the parameters of the mixture and determining the exact number of components that best describes the data set. These issues have been tackled recently by learning the SSDMM via deterministic Maximum Likelihood Estimator (MLE) [22]. Nevertheless, it is known that MLE has major shortcomings linked to its sensitivity at the initialization step. Therefore, a better solution especially for our case (i.e., when dealing with complex medical noisy data including COVID-19 infection) is to develop a more robust alternative based on fully Bayesian inference approach. We recall that Bayesian estimation has attracted a lot of attention for many applications [23,24,25,26,27,28,29,30,31,32,33]. It is also known that the Bayesian approach may be more practical due to the existance of powerful simulation techniques like MCMC [29]. Moreover, the model complexity can be easily solved using for example the marginal likelihood-based technique. Thus, our focus in this paper is to implement an effective Bayesian learning method for SSDMM in order to take into account the complexity of medical data and to overcome the drawbacks of frequentist (deterministic) approaches [34,35]. To the best of our knowledge, such an approach has never been tackled before, especially for the problem of chest x-ray images classification.

The rest of this paper is organized as follows. In next section, the finite shifted-scaled Dirichlet mixture model and the Bayesian approach are exposed. Experimental results and the merits of our approach are introduced in Section 4. Finally, we end this work and provide some possible extensions to be treated in the future.

## 3. Bayesian Framework for the Shifted-Scaled Dirichlet Mixture Model

We start this section by revising both the Dirichlet and scaled Dirichlet distributions, and then introduce a new generalization of these distributions named shifted-scaled Dirichlet distribution (SSDD). The finite shifted-scaled Dirichlet mixture model is also presented. Then, we develop a fully Bayesian framework for learning the parameters of this finite mixture model.

### 3.1. Dirichlet and Scaled-Dirichlet Distributions

**Definition** **1** (Dirichlet distribution). *Let us consider a random vector $Y=\left(y_{1},\ldots ,y_{D}\right)\in S^{D}$ (sample space), where $\sum_{d=1}^{D}y_{d}=1$. We say that Y has a D-variate Dirichlet distribution with parameter $\vec{\alpha }=\left(\alpha_{1},\ldots ,\alpha_{D}\right)\in R_{+}^{D}$ if its density function is:*

(1) $$\begin{array}{cc} Y∼Dir^{D}\left(\vec{\alpha }\right)\\ f\left(\vec{Y}\right)=p\left(\vec{Y}|\theta \right)= & \frac{\Gamma (\alpha_{+})}{\prod_{i=1}^{D}\Gamma \left(\alpha_{i}\right)}\prod_{i=1}^{D}y_{i}^{\alpha_{i}-1} \end{array}$$

*where $\vec{\alpha }$ denotes a shape parameter, $\alpha_{+}=\sum_{i=1}^{D}\alpha_{i}$ and *Γ* indicates the Euler gamma function.*

It is noted that the Dirichlet distribution with D parameters ($Y∼Dir^{D}\left(\vec{\alpha }\right)$) is still popular, especially when it comes to analyzing composition data, and this popularity is due to its its conjugate property with the multinomial likelihood.

**Definition** **2** (Scaled Dirichlet distribution). *If Y follows a scaled Dirichlet distribution, then its density function is given as:*

(2) $$\begin{array}{cc} Y∼SDir^{D}\left(\vec{\alpha },\vec{\beta }\right)\\ f\left(\vec{Y}\right)=p\left(\vec{Y}|\theta \right)= & \frac{\Gamma (\alpha_{+})}{\prod_{i=1}^{D}\Gamma \left(\alpha_{i}\right)}\frac{\prod_{i=1}^{D}\beta_{i}^{\alpha_{i}}y_{i}^{\alpha_{i}-1}}{{\left(\sum_{i=1}^{D}\beta_{i}y_{i}\right)}^{\alpha_{+}}} \end{array}$$

*$\vec{\alpha }=\left(\alpha_{1},\ldots ,\alpha_{D}\right)$ and $\vec{\beta }=\left(\beta_{1},\ldots ,\beta_{D}\right)\in R_{+}^{D}$ are the parameters of this distribution. β is a scale parameter.*

The scaled Dirichlet distribution has 2D parameters and in this case we have $Y∼SDir^{D}\left(\vec{\alpha },\vec{\beta }\right)$. If the parameter β is fixed, then we obtain a Dirichlet model.

### 3.2. Finite Shifted-Scaled Dirichlet Mixture Model

**Definition** **3** (Shifted-Scaled Dirichlet distribution). *Suppose that Y follows a shifted scaled Dirichlet distribution with parameters $\vec{\alpha }=\left(\alpha_{1},\ldots ,\alpha_{D}\right)\in R_{+}^{D}$, $\vec{\lambda }=\left(\lambda_{1},\ldots ,\lambda_{D}\right)\in S^{D}$ and $a\in R_{+}$. Then, the density probability of this distribution is given as:*

(3) $$\begin{array}{cc} Y∼pSDir^{D}\left(\vec{\alpha },\vec{\lambda },a\right)\\ f\left(\vec{Y}\right)=p\left(\vec{Y}|\theta \right)= & \frac{\Gamma (\alpha_{+})}{\prod_{i=1}^{D}\Gamma \left(\alpha_{i}\right)}\frac{1}{a^{D-1}}\frac{\prod_{i=1}^{D}\lambda_{i}^{-(\alpha_{i}/a)}y_{i}^{(\alpha_{i}/a)-1}}{{\left(\sum_{i=1}^{D}{\left(y_{i}/\lambda_{i}\right)}^{\left(1/a\right)}\right)}^{\alpha_{+}}} \end{array}$$

*where $\vec{\lambda }$ denotes a location parameter.*

The shifted-scaled Dirichlet distribution has 2D parameters and in this case we have $Y∼pSDir^{D}\left(\vec{\alpha },\vec{\lambda },a\right)$. If the parameter a=1, then we obtain a scaled Dirichlet model.

Now, suppose that we have a set of vectors $Y=\{{\vec{Y}}_{1},{\vec{Y}}_{2},\ldots ,{\vec{Y}}_{N}\}$, where each vector ${\vec{Y}}_{n}=\left(y_{n1},\ldots ,y_{nD}\right)$ follows a mixture of SSD, then the corresponding likelihood is defined as:

(4) $$p\left(Y|\Theta \right)=\prod_{n=1}^{N}\sum_{k=1}^{K}\pi_{k}p\left({\vec{Y}}_{n}|\theta_{k}\right)$$

where the model’s parameters are defined by $\Theta =(\vec{\pi },\theta )$ and $\left\{\pi_{k}\right\}$ are positive mixing parameters ($\sum_{k}\pi_{k}=1$). Each vector is supposed coming from one component as ${\vec{Y}}_{n}∼pSDir^{D}\left(\vec{\alpha },\vec{\lambda },a\right)$. The shape parameter has the role to describe the form of the shifted SDMM. The scale (*a*) checks how the plotting of the density is distributed and $\vec{\lambda }$ follows the location of the data densities. In the next section, we will develop our Bayesian approach based on the presented mixture of SSDD.

### 3.3. Fully Bayesian Learning Algotithm

In many cases, the deterministic approach (named also maximum likelihood-based technique) via the well known EM algorithm [36] is used to estimate the parameters of finite mixture models due to its simplicity. Deterministic approach assumes that $Z=({\vec{Z}}_{1},\ldots ,{\vec{Z}}_{N})$, is a missing data. Thus, if ${\vec{Y}}_{n}\in j$ then $Z_{ij}=1$, else $Z_{nj}=0$. Because the likelihood-technique depends on initial values and is sensitive to local minima, we propose here to overcome these limitations by developing an efficient way based on Bayesian inference to better learn the Shifted-Scaled Dirichlet mixture model. More precisely, we propose to investigate one of the effective simulation techniques called Markov Chain Monte Carlo (MCMC) via Gibbs sampler [37,38]. Thus, the complete likelihood is defined as:

(5) $$p\left(Y,Z|\Theta \right)=\prod_{n=1}^{N}\prod_{k=1}^{K}{\left(\pi_{k}p\left({\vec{Y}}_{n}|\theta_{k}\right)\right)}^{Z_{nk}}$$

Using Bayes formula, the likelihood and the priors will be expressed together to define the posterior distribution like this:

(6) p(Θ|Y,Z)∝p(Y,Z|Θ)p(Θ)

The proposed Bayesian algorithm for SSDMM parameters’ learning is based on the following steps:

1. Initialization
2. Step t: For t = 1,…
  (a) Generate ${\vec{Z}}_{i}^{\left(t\right)}∼M\left(1;{\hat{Z}}_{i1}^{\left(t-1\right)},\ldots ,{\hat{Z}}_{iM}^{\left(t-1\right)}\right)$
  (b) Generate ${\vec{\pi }}^{\left(t\right)}$ from $p(\pi |Z^{\left(t\right)})$
  (c) Generate ${\left(\theta \right)}^{\left(t\right)}$ from $p(\theta |Z^{\left(t\right)},Y)$

where $M(1;{\hat{Z}}_{i1}^{\left(t-1\right)},\ldots ,{\hat{Z}}_{iM}^{\left(t-1\right)})$ is a multinomial distribution of order one with parameters $\left(p{\left(1|{\vec{Y}}_{i}\right)}^{\left(t-1\right)},\ldots ,p{\left(M|{\vec{Y}}_{i}\right)}^{\left(t-1\right)}\right)$. Based on this algorithm, we have to evaluate p(π|Z) and p(θ|Z,Y).

#### 3.3.1. Priors and Posteriors

The choice of priors is one of the most crucial steps in Bayesian modeling. These priors reflect our belief about the the model’s parameters and are updated and enhanced according to the observed data (see for example details in [39]). In the following, the choice of the priors is addressed as well as the determining of the resulting posteriors for our fully Bayesian approach.

Estimating the posterior will lead to have our parameters Θ∼p(Θ|Y,Z). In order to perform this step, we proceed with an elegant sampling technique called Gibbs sampler. This method allows the use of conditional posterior distribution in order to update each parameter.

Since no convenient conjugate prior exist for ${\vec{\alpha }}_{k}$ and $a_{k}$, we adopt a common choice for them which is the Gamma distribution G(.):

(7) $$p\left(\alpha_{kd}\right)=G\left(\alpha_{kd}|u_{kd},v_{kd}\right)$$

(8) $$p\left(a_{k}\right)=G\left(a_{k}|g_{k},h_{k}\right)$$

Then, we determine the posterior distributions according to these priors and by considering the following:

(9) $$p\left({\vec{\alpha }}_{k}|Z,Y\right)\propto p\left({\vec{\alpha }}_{k}\right)\prod_{Z_{ik}=1}p\left({\vec{Y}}_{i}|\theta_{k}\right)\propto \prod_{d=1}^{D}p\left(\alpha_{kd}\right)\prod_{Z_{ik}=1}p\left({\vec{Y}}_{i}|\theta_{k}\right)$$

(10) $$p\left(a_{k}|Z,Y\right)\propto p\left(a_{k}\right)\prod_{Z_{ik}=1}p\left({\vec{Y}}_{i}|\theta_{k}\right)$$

Regarding the parameter ${\vec{\lambda }}_{k}$, since it is defined in a simplex, therefore, it is a common and classic choice in Bayesian inference to choose the Dirichlet distribution as prior with parameters $\eta_{k}=\left(\eta_{k1},\ldots ,\eta_{kD}\right)$. So, it is expressed as:

(11) $$p\left({\vec{\lambda }}_{k}|\eta_{k}\right)=\frac{\Gamma (\sum_{j=1}^{D}\eta_{kj})}{\prod_{j=1}^{D}\Gamma \left(\eta_{kj}\right)}\prod_{j=1}^{D}p_{kj}^{\eta_{kj}-1}$$

Knowing this prior, we can estimate the posterior distribution using the following equation:

(12) $$p\left({\vec{\lambda }}_{k}|Z,Y\right)\propto p\left({\vec{\lambda }}_{k}|\eta_{k}\right)\prod_{Z_{ik}=1}p\left({\vec{Y}}_{i}|\theta_{k}\right)$$

For the prior of mixing weight $\vec{\pi }$, the common choice is the Dirichlet distribution since $\sum_{j=1}^{K}\pi_{j}=1$. So, the mixing weight prior is expressed as:

(13) $$p\left(\vec{\pi }|K,\delta \right)=\frac{\Gamma (\sum_{j=1}^{K}\delta_{j})}{\prod_{j=1}^{K}\Gamma \left(\delta_{j}\right)}\prod_{j=1}^{K}\pi_{j}^{\delta_{j}-1}$$

The selected prior of Z ( membership variable ) is defined as:

(14) $$p\left(Z|\vec{\pi },K\right)=\prod_{j=1}^{K}\pi_{j}^{n_{j}}$$

where $n_{j}$ is the tiotal vectors in cluster *j*. Given the former equations Equations (13) and (14) we have

(15) $$\begin{array}{ccc} p(\vec{\pi }|\ldots ) & \propto & p\left(Z|\vec{\pi },K\right)p\left(\vec{\pi }|K,\delta \right)\\ & \propto & \prod_{j=1}^{K}\pi_{j}^{n_{j}}\frac{\Gamma (\sum_{j=1}^{K}\delta_{j})}{\prod_{j=1}^{K}\Gamma \left(\delta_{j}\right)}\prod_{j=1}^{K}\pi_{j}^{\delta_{j}-1}\propto \frac{\Gamma (\sum_{j=1}^{K}\delta_{j})}{\prod_{j=1}^{K}\Gamma \left(\delta_{j}\right)}\prod_{j=1}^{K}\pi_{j}^{n_{j}+\delta_{j}-1} \end{array}$$

This posterior is proportional to the Dirichlet distribution $\left(\delta_{1}+n_{1},\ldots ,\delta_{K}+n_{K}\right)$. In addition, the posterior of the membership Z may be deduced as:

(16) $$p\left(Z_{i}=j|\ldots \right)\propto \pi_{j}p\left({\vec{Y}}_{n}|\theta_{j}\right)$$

**Figure 1:**
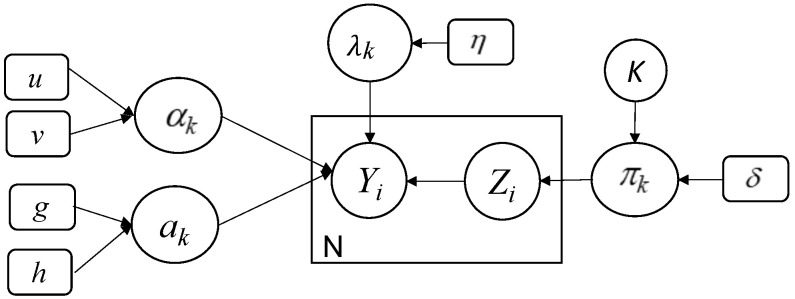
Graphical representation of our developed Bayesian finite shifted-scaled Dirichlet mixture model. Fixed hyperparameters are indicated by rounded boxes and random variables by circles. *Y* is the observed variable, *Z* represents the latent variable, the large box indicates repeated process, and the arcs show the dependencies between variables.

Finally, we choose the uniform distribution as an appropriate prior for *K*. This value can vary between 1 and $K_{max}$ ($K_{max}$ is a predefined value). We summarize the proposed model in the following graphical representation Figure 1.

#### 3.3.2. Complete Bayesian Estimation-Algorithm

The Gibbs sampling technique is mainly based on alternating conditional distributions for several steps. Indeed, for each iteration *t*, the resulted estimate $\Theta^{t}$ is sampled from its previous approximate $\Theta^{t-1}$. Having all these posterior probabilities in hand, the complete MCMC-based Bayesian algorithm to learn the parameters of our finite mixture model and especially the steps of our Gibbs sampler are as follows:

1. Initialization
2. Step t: For t = 1,…
  (a) Generate $Z_{i}^{\left(t\right)}∼M\left(1;{\hat{Z}}_{i1}^{\left(t-1\right)},\ldots ,{\hat{Z}}_{iK}^{\left(t-1\right)}\right)$
  (b) Compute $n_{k}^{\left(t\right)}=\sum_{i=1}^{N}I_{Z_{ik}^{\left(t\right)}=j}$
  (c) Generate ${\vec{\pi }}^{\left(t\right)}$ from Equation (15)
  (d) Generate ${\vec{\alpha }}_{k}^{\left(t\right)}$, $a_{k}^{\left(t\right)}$, and ${\vec{\lambda }}_{k}^{\left(t\right)}$(k=1,…,K) from Equations (9), (10) and (12), respectively, using random-walk Metropolis-Hastings (M-H) algorithm [40,41].

where $M(1;{\hat{Z}}_{i1}^{\left(t-1\right)},\ldots ,{\hat{Z}}_{iM}^{\left(t-1\right)})$ is a multinomial distribution of order one with parameters $\left(p{\left(1|{\vec{Y}}_{i}\right)}^{\left(t-1\right)},\ldots ,p{\left(M|{\vec{Y}}_{i}\right)}^{\left(t-1\right)}\right)$.

## 4. Experimental Results

The goal of this section is to evaluate and validate the developed statistical model with the different inference techniques. We have considered several real data sets of images including COVID-19 and different pneumonia types.

### 4.1. Data Sets

**Figure 2:**
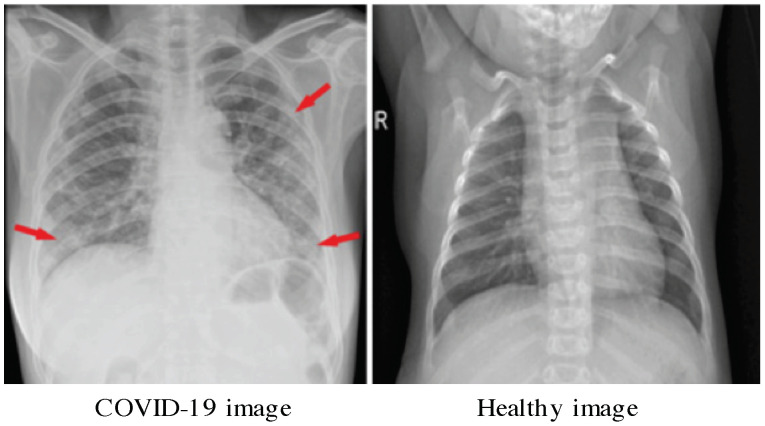
Illustrative sample of Chest X-Rays image with COVID-19 [43].

The first main COVID-19dataset (https://github.com/ieee8023/covid-chestxray-dataset) for our experiments is the one developed by Cohen et al. [42]. It contains 542 Chest X-ray (CXR) images. A subset of 434 CXR images represent patients positive to COVID-19 and the rest are COVID-19 negative. The image dimension is 4248 × 3480 pixels. Main statistics of this dataset are given in Table 1. An illustrative sample of confirmed Coronavirus Disease 2019 (COVID-19) is given in Figure 2. This image is from a 53-year-old female who had a fever and cough for 5 days. Indeed, Multifocal patchy opacities can be seen in both lungs (arrows) [43].

**Figure 3:**
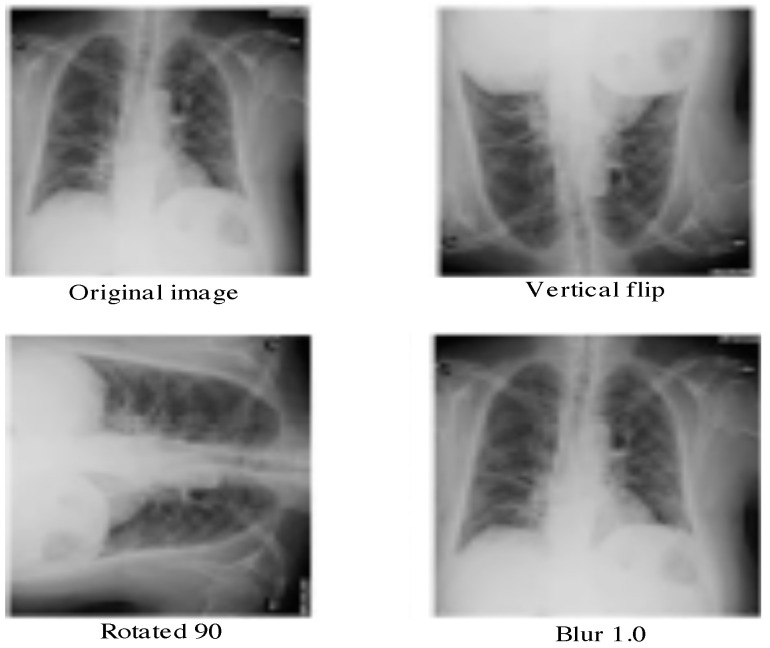
Illustrative examples of augmented Chest X-Rays with COVID-19 from the dataset [44].

We run also our implemented framework on another available dataset named Augmented COVID-19 Dataset (https://data.mendeley.com/datasets/2fxz4px6d8/4). It is collected from the previous dataset and the Kaggle one (kaggle.com/paultimothymooney/chest-xray-pneumonia). It is made up of augmented radiographics with and without COVID-19. Here, the number of images is larger than the previous dataset. Our aim is to study the performance of our model when the size of the data increases. This dataset contains 912 COVID-19 images and 912 non COVID-19 images. The augmentation process takes into account some geometric transformations and other ones such as translation, rotation, scaling, flipping, noising, bluring, etc. Some illustrative augmented images are given in Figure 3.

**Figure 4:**
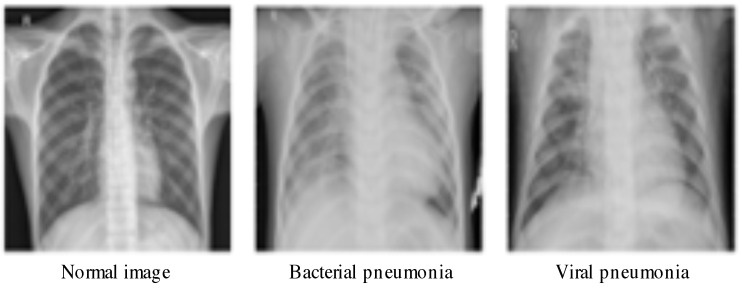
Illustrative samples of chest-xray-pneumonia from the dataset in [45].

Finally, we use the chest-xray-pneumonia to evaluate the performance. Thus, we rum our algorithm on big dataset (viral, bacterial infection, and normal) Kaggle (https://www.kaggle.com/paultimothymooney/chest-xray-pneumonia). It contains 5856 CXR images where 1583 are normal and 4273 are infected with pneumonia. The image dimension is 1024 × 1024 pixels. This dataset is structured into three folders: train, test and val. Some samples are given in Figure 4. Statistics about this dataset are shown in Table 1.

### 4.2. Methodology

**Figure 5:**
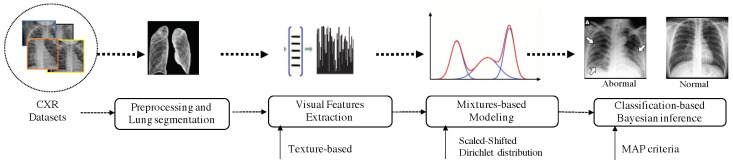
The pipeline of the proposed method. First, the lungs are segmented, then robust visual features are extracted. Features are modelled using the proposed mixture model (SSDDMM) and a Bayesian framework is applied to estimate the parameters of the model. Finally, images are classified on the basis of Bayes rule.

The developed model is applied to classify several images from different datasets as normal or COVID-19 affected patients using CXR images. To deal with this objective, we proceed with some preprocessing steps. After a pre-segmentation step of the lung region, we extracted some relevant features based on texture analysis. Indeed, several recently published works have shown that the lung is the basic organ which is affected by the corona COVID-19 virus. The classification is performed into two classes: normal and abnormal. Each image is modelled with a mixture of SSDDMM, then we apply the MCMC algorithm to estimate the parameters of each component. Here, the classification problem is presented in terms of assigning each image to the appropriate class using the Bayes rules. In other word, each image is affected to the class that has the greatest posterior probability. The pipeline of the proposed method is given in Figure 5.

**Figure 6:**
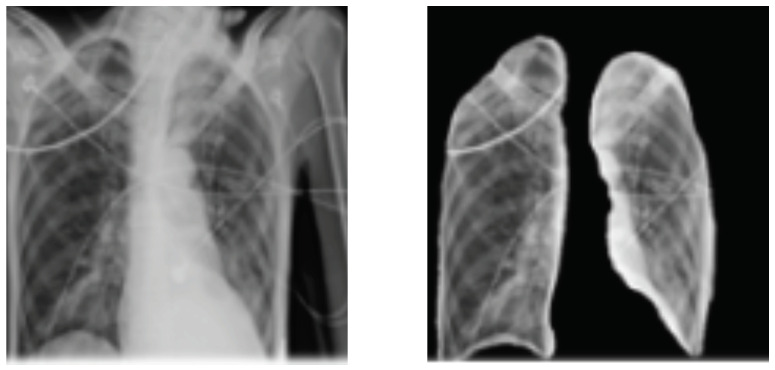
Process of lungs regions extraction applied on image sample from [42].

It is noted that, in many cases, medical images such as chest x-rays are not easy to interpret; thus, it is mandatory to identify important patterns to interpret better and improve the decision. Feature extraction problem is the process of acquiring relevant information such as texture. The step of feature extraction has the role to improve the performance and accelerate the processing time. In particular, texture’s structures (e.g., fine, smooth, coarse or grained) characterize effectively visual patterns in the image. In the state of the art, many texture extraction methods have been proposed such as statistical ones which are based on different statistics order of the gray-level value. For complex images like medical ones, the use of single feature value cannot lead to satisfactory results; thus, it is important to consider more features to increase the expected performance [46]. In this work, we focus on investigating the so-called Gray Level Co-occurrence Matrix (GLCM)-based features, which has been shown to be efficient and offer interesting results in term of classification accuracy. GLCM matrix provides a co-occurrence matrix of joint probability density of the gray levels of two pixels. In this work, the second-order statistics are investigated to compute some features in order to well-discriminate lung abnormalities. In particular, the following features [47] are calculated for each image: contrast (large differences between neighboring pixels), correlation, energy, entropy, difference variance, difference entropy, inverse difference normalized, information measure of correlation, information measure of correlation. In our analysis, we focused on extracting the lungs area using image thresholding and segmentation processing which leads to identify the left and right lungs from CXR images. In order to remove noise, we applied the Gaussian filter. In Figure 6, we illustrate the obtained segmented lung using the above method. After isolating the lungs, we proceed with feature extraction step and then with classification using the proposed statistical model. The required time for feature extraction for each image is a few seconds and the model fitting taken between 20 to 30 min for the different data sets.

### 4.3. Results Analysis

In this section, we investigate our approach for COVID-19 detection. The ultimate first goal is to prove the potential of our Bayesian learning algorithm as compared to other learning method named maximum likelihood (ML) estimation. The second goal is to compare the performance of the proposed shifted-scaled Dirichlet mixture model with other methods which are Gaussian mixture-based, Gamma mixture-based, Dirichlet mixture-based and scaled Dirichlet mixture-based method. For performance investigation, we evaluate the performance of our Bayesian learning method and the rest of methods in terms of overall accuracy (ACC), detection rate (DR), and false-positive rate (FPR). Table 2, Table 3 and Table 4, show the classification accuracies for the Test sets of each dataset when applying different generative approaches namely: Gaussian mixture model with maximum likelihood (**GMM-ML**), with Bayesian inference (**GMM-B**), Gamma mixture model with maximum likelihood (**ΓMM-ML**), Dirichlet mixture with maximum likelihood (**DMM-ML**), with Bayesian inference (**DMM-B**), scaled Dirichlet mixture with maximum likelihood (**SDMM-ML**), with Bayesian inference (**SDMM-B**), shifted scaled Dirichlet mixture with maximum likelihood (**SSDMM-ML**), and our proposed method named as shifted scaled Dirichlet mixture with Bayesian inference (**SSDMM-B**).

According to these tables, we can see clearly that, in general, all mixture models provide encouraging results taking into account the difficulty of the unsupervised learning problem. It is clear that our proposed Bayesian method for the shifted scaled Dirichlet mixture outperforms, according to the used metrics, the rest of methods. Indeed, our work has better accuracy as well as lowest false positive rate than both Dirichlet and Gaussian mixtures. We can also see that Bayesian learning provides better results than the ML approach for all models. As we can see, for CXR-COVID dataset, the SSDMM-B outperforms other models with accuracy of 89.57% compared to 88.08% for SDMM-B, 88.04% for DMM and 82.44% for GMM. Our Bayesian model is slightly better than SSDMM-MML [22]. Likewise, we came to the same conclusion for the other datasets and we reach the highest accuracy of 93.03% with our model **SSDMM-B** for the CXR-Pneumonia dataset. According to this last result, it is clear that the precision increases (and the false positive decreases) as the dataset size increases. This is can be viewed for CXR-Augmented-COVID and CXR-Pneumonia datasets which contain more images than CXR-COVID. On the basis of the overall accuracy (ACC) for three datasets (CXR-COVID, CXR-Pneumonia, and CXR-Augmented-COVID), it is obviously clear that the difference between the highest and lowest accuracy is between 5.2% and 7.46% for each dataset. The difference between some methods is about 2.26% which is also considered significant according to t-student test. The obtain results confirm the merits of the fully Bayesian formalism for shifted-scaled Dirichlet mixture which is more flexible (since it has more degrees of freedom) than the Dirichlet and the scaled Dirichlet mixtures. Its flexibility also makes it possible to easily integrate more knowledge and especially features selection mechanism into the proposed framework. On the other hand, even a small improvement is worthwhile taking into account the difficulty of the problem especially with the availability of strong machines to do the processing and simulations. Concerning the modeling uncertainty quantification, this is something that distinguishes our approach from deep learning models (black boxes). We are currently working with clinicians to be able to quantify the uncertainty and extract interpretations, as well as explanations from our models which is possible thanks to the generative nature of the deployed model.

It is also noted that the lung segmentation step is difficult particularly when it includes acute respiratory distress syndrome. This difficulty is due to the little contrast at the boundary of the lung. Moreover, when the number of images in this dataset is too small, the obtained results are lower than the case of big datasets. We can conclude that the obtained results are considered very encouraging given that we approach the classification problem in an unsupervised manner. In fact, the flexibility of the shifted-scaled mixture model and the robustness of texture-based features lead to more stable results. For COVID-19 identification through CXR images, the proposed fully Bayesian learning approach for SSDMM has confirmed that it is capable to discriminate images according to texture properties. In order to further improve these results, perhaps other descriptors are needed, especially the consideration of a robust feature selection mechanism to filter out unreliable features and keep only the most relevant ones. Please note that various studies have been proposed in the state of the art [53] which show that textures are very promising for many medical applications [54]. Here, the comparison between different feature-based techniques is beyond the scope of this article. Instead, we investigated in this work one robust texture-based descriptor to have interesting results for the classification of chest x-ray (CXR) images and corona virus convid-19 detection.

## 5. Conclusions

In this paper, we have addressed the problems of modeling and classification of multidimensional non-Gaussian data via a purely Bayesian learning approach based on a shifted scaled Dirichlet mixture model. We have especially tackled the problems of chest x-ray (CXR) images classification and COVID-19 detection. The flexibility and capability of the proposed statistical framework is evaluated through three public datasets related to COVID-19 and Pneumonia diseases. Unlike other statistical methods, which assume the heavy assumption that input data are Gaussian, which is not always ture especially for real medical applications, the treated data in our work are modelled via non-Gaussian model and using finite mixtures of shifted scaled Dirichlet distributions that offer reasonable explanations. Our framework has provided promising results and outperforms other methods. In particular, the Bayesian inference results are more interesting thanks to the consideration of the joint posterior distribution. In this work we have investigated an effective MCMC-based approximation technique given that exact inference in fully Bayesian methods is not easy to compute. Our implemented approach has also the advantage of being more general and extensible enough to be applied for large scale data presenting various infection’s type. Future works could be devoted to extending the proposed framework via nonparametric approaches. Other promising future works include the integration of feature selection mechanism into the statistical model to improve the generalization capabilities. We hope also that many other real-world problems, including medical ones, will be addressed within the proposed framework.

## Figures and Tables

**Table 1:** Data description.

Dataset	Class	Train	Validation	Test	Total
CXR-COVID	Non-COVID-19	70	20	18	108
	COVID-19	328	80	26	434
CXR-Augmented-COVID	Non-COVID-19	512	100	300	912
	COVID-19	512	100	300	912
CXR-Pneumonia	Normal	1341	8	234	1583
	Pneumonia	3875	8	390	4273

**Table 2:** Overall accuracy for chest x-ray (CXR)-COVID Dataset.

Approach/Metrics	ACC(%)	DR(%)	FPR(%)
**GMM-ML** [48]	82.11	81.02	0.18
**GMM-B** [49]	83.44	82.14	0.17
**ΓMM-ML** [50]	85.22	83.76	0.16
**DMM-ML** [51]	87.99	87.88	0.14
**DMM-B** [52]	88.04	87.78	0.13
**SDMM-ML** [16]	88.08	87.84	0.13
**SDMM-B** [31]	88.22	88.07	0.13
**SSDMM-ML** [22]	89.13	88.24	0.12
**SSDMM-B (our method)**	**89.57**	**88.61**	**0.12**

**Table 3:** Overall accuracy for CXR-Pneumonia Dataset.

Approach/Metrics	ACC(%)	DR(%)	FPR(%)
**GMM-ML** [48]	87.66	85.80	0.13
**GMM-B** [49]	88.90	86.98	0.11
**ΓMM-ML** [50]	90.54	88.54	0.10
**DMM-ML** [51]	91.81	91.03	0.09
**DMM-B** [52]	92.01	91.33	0.09
**SDMM-ML** [16]	92.43	91.32	0.09
**SDMM-B** [31]	92.81	91.77	0.09
**SSDMM-ML** [22]	92.85	92.01	0.08
**SSDMM-B (our method)**	**93.03**	**92.90**	**0.08**

**Table 4:** Overall accuracy for CXR-Augmented COVID-19 Dataset.

Approach/Metrics	ACC(%)	DR(%)	FPR(%)
**GMM-ML** [48]	85.13	83.99	0.14
**GMM-B** [49]	86.77	84.08	0.13
**ΓMM-ML** [50]	90.24	89.14	0.10
**DMM-ML** [51]	88.01	87.57	0.12
**DMM-B** [52]	88.44	87.96	0.12
**SDMM-ML** [16]	89.01	88.12	0.11
**SDMM-B** [31]	89.88	89.12	0.10
**SSDMM-ML** [22]	90.10	89.01	0.09
**SSDMM-B (our method)**	**90.33**	**89.12**	**0.09**

## Data Availability

https://github.com/ieee8023/covid-chestxray-dataset; https://www.kaggle.com/paultimothymooney/chest-xray-pneumonia; https://data.mendeley.com/datasets/2fxz4px6d8/4.

## References

[1] Jacobi A., Chung M., Bernheim A., Eber C. (2020). Portable chest X-ray in coronavirus disease-19 (COVID-19): A pictorial review. Clin. Imaging.

[2] Parveen N., Sathik M.M. (2011). Detection of pneumonia in chest X-ray images. J. X-ray Sci. Technol..

[3] Ginneken B.V., Stegmann M.B., Loog M. (2006). Segmentation of anatomical structures in chest radiographs using supervised methods: A comparative study on a public database. Med. Image Anal..

[4] Minaee S., Kafieh R., Sonka M., Yazdani S., Soufi G.J. (2020). Deep-COVID: Predicting COVID-19 from chest X-ray images using deep transfer learning. Med. Image Anal..

[5] Gordienko Y., Gang P., Hui J., Zeng W., Kochura Y., Alienin O., Rokovyi O., Stirenko S. (2018). Deep learning with lung segmentation and bone shadow exclusion techniques for chest x-ray analysis of lung cancer. International Conference on Computer Science, Engineering and Education Applications.

[6] Oliveira L.L.G., e Silva S.A., Ribeiro L.H.V., de Oliveira R.M., Coelho C.J., Andrade A.L.S.S. (2008). Computer-aided diagnosis in chest radiography for detection of childhood pneumonia. Int. J. Med. Inform..

[7] Litjens G., Kooi T., Bejnordi B.E., Setio A.A.A., Ciompi F., Ghafoorian M., van der Laak J.A.W.M., van Ginneken B., Sánchez C.I. (2017). A survey on deep learning in medical image analysis. Med. Image Anal..

[8] Greenspan H., van Ginneken B., Summers R.M. (2016). Guest Editorial Deep Learning in Medical Imaging: Overview and Future Promise of an Exciting New Technique. IEEE Trans. Med. Imaging.

[9] Zhao B., Feng J., Wu X., Yan S. (2017). A survey on deep learning-based fine-grained object classification and semantic segmentation. Int. J. Autom. Comput..

[10] Novikov A.A., Lenis D., Major D., Hladůvka J., Wimmer M., Bühler K. (2018). Fully convolutional architectures for multiclass segmentation in chest radiographs. IEEE Trans. Med Imaging.

[11] Xu X., Jiang X., Ma C., Du P., Li X., Lv S., Yu L., Chen Y., Su J., Lang G. (2020). Deep Learning System to Screen Coronavirus Disease 2019 Pneumonia. Engineering.

[12] Da Nóbrega R.V.M., Filho P.P.R., Rodrigues M.B., da Silva S.P.P., Júnior C.M.J.M.D., de Albuquerque V.H.C. (2020). Lung nodule malignancy classification in chest computed tomography images using transfer learning and convolutional neural networks. Neural Comput. Appl..

[13] Candemir S., Jaeger S., Palaniappan K., Musco J.P., Singh R.K., Xue Z., Karargyris A., Antani S., Thoma G., McDonald C.J. (2014). Lung Segmentation in Chest Radiographs Using Anatomical Atlases With Nonrigid Registration. IEEE Trans. Med. Imaging.

[14] Xu T., Mandal M.K., Long R., Cheng I., Basu A. (2012). An edge-region force guided active shape approach for automatic lung field detection in chest radiographs. Comput. Med. Imaging Graph..

[15] Mashrgy M.A., Bdiri T., Bouguila N. (2014). Robust simultaneous positive data clustering and unsupervised feature selection using generalized inverted Dirichlet mixture models. Knowl. Based Syst..

[16] Oboh B.S., Bouguila N. Unsupervised learning of finite mixtures using scaled dirichlet distribution and its application to software modules categorization. Proceedings of the 2017 IEEE International Conference on Industrial Technology (ICIT).

[17] Channoufi I., Bourouis S., Bouguila N., Hamrouni K. (2018). Image and video denoising by combining unsupervised bounded generalized gaussian mixture modeling and spatial information. Multimed. Tools Appl..

[18] Fan W., Bouguila N. (2020). Spherical data clustering and feature selection through nonparametric Bayesian mixture models with von Mises distributions. Eng. Appl. Artif. Intell..

[19] Najar F., Bourouis S., Bouguila N., Belghith S. (2019). Unsupervised learning of finite full covariance multivariate generalized Gaussian mixture models for human activity recognition. Multimed. Tools Appl..

[20] Najar F., Bourouis S., Zaguia A., Bouguila N., Belghith S. Unsupervised Human Action Categorization Using a Riemannian Averaged Fixed-Point Learning of Multivariate GGMM. Proceedings of the Image Analysis and Recognition-15th International Conference, ICIAR.

[21] Bourouis S., Mashrgy M.A., Bouguila N. (2014). Bayesian learning of finite generalized inverted Dirichlet mixtures: Application to object classification and forgery detection. Expert Syst. Appl..

[22] Alsuroji R., Zamzami N., Bouguila N. Model Selection and Estimation of a Finite Shifted-Scaled Dirichlet Mixture Model. Proceedings of the 17th IEEE International Conference on Machine Learning and Applications, ICMLA.

[23] Alroobaea R., Rubaiee S., Bourouis S., Bouguila N., Alsufyani A. (2020). Bayesian inference framework for bounded generalized Gaussian-based mixture model and its application to biomedical images classification. Int. J. Imaging Syst. Technol..

[24] Kayabol K., Kutluk S. (2016). Bayesian classification of hyperspectral images using spatially-varying Gaussian mixture model. Digit. Signal Process..

[25] Li Z., Xia Y., Ji Z., Zhang Y. (2017). Brain voxel classification in magnetic resonance images using niche differential evolution based Bayesian inference of variational mixture of Gaussians. Neurocomputing.

[26] Li F., Perona P. A Bayesian Hierarchical Model for Learning Natural Scene Categories. Proceedings of the 2005 IEEE Computer Society Conference on Computer Vision and Pattern Recognition (CVPR 2005).

[27] Bourouis S., Al-Osaimi F.R., Bouguila N., Sallay H., Aldosari F.M., Mashrgy M.A. (2019). Bayesian inference by reversible jump MCMC for clustering based on finite generalized inverted Dirichlet mixtures. Soft Comput..

[28] Robert C. (2007). The Bayesian Choice: From Decision-Theoretic Foundations to Computational Implementation.

[29] Marin J.M., Robert C. (2007). Bayesian Core: A Practical Approach to Computational Bayesian Statistics.

[30] Chen P., Nelson J.D.B., Tourneret J. (2017). Toward a Sparse Bayesian Markov Random Field Approach to Hyperspectral Unmixing and Classification. IEEE Trans. Image Process..

[31] Bourouis S., Laalaoui Y., Bouguila N. (2019). Bayesian frameworks for traffic scenes monitoring via view-based 3D cars models recognition. Multimed. Tools Appl..

[32] Barber D., Williams C.K.I., Mozer M., Jordan M.I., Petsche T. (1996). Gaussian Processes for Bayesian Classification via Hybrid Monte Carlo. Proceedings of the Advances in Neural Information Processing Systems 9, NIPS.

[33] Bourouis S., Al-Osaimi F.R., Bouguila N., Sallay H., Aldosari F.M., Mashrgy M.A. Video Forgery Detection Using a Bayesian RJMCMC-Based Approach. Proceedings of the 14th IEEE/ACS International Conference on Computer Systems and Applications, AICCSA 2017.

[34] Fan W., Bouguila N., Bourouis S., Laalaoui Y. (2018). Entropy-based variational Bayes learning framework for data clustering. IET Image Process..

[35] Bourouis S., Zaguia A., Bouguila N., Campilho A., Karray F., ter Haar Romeny B.M. (2018). Hybrid Statistical Framework for Diabetic Retinopathy Detection. Image Analysis and Recognition, Proceedings of the 15th International Conference, ICIAR 2018, Póvoa de Varzim, Portugal, 27–29 June 2018.

[36] Dempster A.P., Laird N.M., Rubin D.B. (1977). Maximum likelihood from incomplete data via the EM algorithm. J. R. Stat. Soc. Ser. B.

[37] Bouguila N. (2011). Bayesian hybrid generative discriminative learning based on finite Liouville mixture models. Pattern Recognit..

[38] Gelman A., Carlin J.B., Stern H.S., Rubin D.B. (2013). Bayesian Data Analysis.

[39] Geiger D., Heckerman D. Parameter priors for directed acyclic graphical models and the characterization of several probability distributions. Proceedings of the Fifteenth Conference on Uncertainty in Artificial Intelligence.

[40] Congdon P. (2003). Applied Bayesian Modelling.

[41] Chib S., Greenberg E. (1995). Understanding the Metropolis-Hastings Algorithm. Am. Stat..

[42] Cohen J.P., Morrison P., Dao L., Roth K., Duong T.Q., Ghassemi M. (2020). COVID-19 Image Data Collection: Prospective Predictions Are the Future. arXiv.

[43] Zu Z.Y., Jiang M.D., Xu P.P., Chen W., Ni Q.Q., Lu G.M., Zhang L.J. (2020). Coronavirus disease 2019 (COVID-19): A perspective from China. Radiology.

[44] Alqudah A., Qazan S. (2020). Augmented COVID-19 X-ray images dataset. Mendeley Data.

[45] Mooney P. (2020). Chest X-ray Images (Pneumonia). https://www.kaggle.com/paultimothymooney/chest-xray-pneumonia.

[46] Xie J., Jiang Y., Tsui H. (2005). Segmentation of kidney from ultrasound images based on texture and shape priors. IEEE Trans. Med. Imaging.

[47] Pourghassem H., Ghassemian H. (2008). Content-based medical image classification using a new hierarchical merging scheme. Comput. Med. Imaging Graph..

[48] Fernando B., Fromont É., Muselet D., Sebban M. (2012). Supervised learning of Gaussian mixture models for visual vocabulary generation. Pattern Recognit..

[49] Figueiredo M.A.T., Jain A.K. (2002). Unsupervised Learning of Finite Mixture Models. IEEE Trans. Pattern Anal. Mach. Intell..

[50] Sallay H., Bourouis S., Bouguila N. (2021). Online Learning of Finite and Infinite Gamma Mixture Models for COVID-19 Detection in Medical Images. Computers.

[51] Bouguila N., Ziou D. (2005). Using unsupervised learning of a finite Dirichlet mixture model to improve pattern recognition applications. Pattern Recognit. Lett..

[52] Ma Z., Rana P.K., Taghia J., Flierl M., Leijon A. (2014). Bayesian estimation of Dirichlet mixture model with variational inference. Pattern Recognit..

[53] Smith G., Burns I. (1997). Measuring texture classification algorithms. Pattern Recognit. Lett..

[54] Melendez J., van Ginneken B., Maduskar P., Philipsen R.H.H.M., Reither K., Breuninger M., Adetifa I.M.O., Maane R., Ayles H., Sánchez C.I. (2015). A Novel Multiple-Instance Learning-Based Approach to Computer-Aided Detection of Tuberculosis on Chest X-Rays. IEEE Trans. Med. Imaging.

